# Poly(vinylbenzyl Pyridinium Salts) as Novel Sorbents for Hazardous Metals Ions Removal

**DOI:** 10.3390/molecules27051723

**Published:** 2022-03-06

**Authors:** Karolina Wieszczycka, Kinga Filipowiak, Aneta Lewandowska, Agnieszka Marcinkowska, Marek Nowicki

**Affiliations:** 1 Faculty of Chemical Technology, Institute of Chemical Technology and Engineering, Poznan University of Technology, Berdychowo 4, 60-965 Poznan, Poland; kinga.m.filipowiak@doctorate.put.poznan.pl (K.F.); aneta.b.lewandowska@doctorate.put.poznan.pl (A.L.); agnieszka.marcinkowska@put.poznan.pl (A.M.); 2 Faculty of Materials Engineering and Technical Physics, Institute of Physics, Poznan University of Technology, Piotrowo 3, 60-965 Poznan, Poland; marek.nowicki@put.poznan.pl; 3 Center for Advanced Technology, Adam Mickiewicz University, Uniwersytetu Poznańskiego 10, 61-614 Poznan, Poland

**Keywords:** resins, Pb(II), Cd(II), wastewaters, sorption

## Abstract

Novel efficient complexing resins—poly(vinylbenzyl pyridinium salts) fabricated through poly(vinylbenzyl halogene-co-divinylbenzene) quaternization of *N*-decyloxy-1-(pyridin-3-yl)ethaneimine and *N*-decyloxy-1-(pyridin-4-yl)ethaneimine—were tested as adsorbents of Pb(II), Cd(II), Cu(II), Zn(II), and Ni(II) from aqueous solutions. The structure of these materials was established by ^13^C CP-MAS NMR, X-ray photoelectron spectroscopy, elemental analysis, and Fourier transform infrared spectroscopy, as well as thermogravimetric and differential thermal analyses. The textural properties were determined using scanning electron microscopy and low-temperature N_2_ sorption. Based on the conducted sorption studies, it was shown that the uptake behavior of the metal ions towards novel resins depended on the type of functionalities, contact time, pH, metal concentrations, and the resin dosage. The Langmuir model was investigated to be the best one for fitting isothermal adsorption equilibrium data, and the corresponding adsorption capacities were predicted to be 296.4, 201.8, 83.8, 38.1, and 39.3 mg/g for Pb(II), Zn(II), Cd(II), Cu(II), and Ni(II), respectively. These results confirmed that owing to the presence of the functional pyridinium groups, the resins demonstrated proficient metal ion removal capacities. Furthermore, VBBr-D4EI could be successfully used for the selective uptake of Pb(II) from wastewater. It was also shown that the novel resins can be regenerated without significant loss of their sorption capacity.

## 1. Introduction

Industrial development, and thus the production of various types of materials and devices, is necessary, but it also involves waste that may cause irreversible changes in the natural environment. Especially, heavy metal pollution in water bodies or groundwater poses a major threat to all living forms [1,2,3]. The presence of heavy metal ions in the environment is mainly caused by the production of wastewater by plants producing paper, fabrics, detergents, and food, but also to a large extent from the chemical, metal refining, petroleum, and petrochemical industries, as well as pharmaceuticals [4]. Some of the heavy metal ions have a positive effect on the functioning of living organisms; for example, zinc is a macroelement that affects the immune system and proper insulin secretion, and also increases sperm production, while copper is a micronutrient involved in the synthesis of collagen and elastin. However, when the permissible concentrations are exceeded, the ions of these metals become toxic. For example, lead tends to accumulate in the blood and soft tissues, which can cause hemoglobin synthesis disorders; damage to the kidneys, liver, lungs, and brain; and even lead to mental retardation and abnormalities in pregnant women [1]. Due to their toxicity and non-biodegradability, effective and selective techniques for the elimination of toxic metals are widely sought [5,6]. The processes for removing heavy metals from wastewater are based on various methods such as chemical precipitation, flotation, ion exchange, and electrochemical deposition, as well as membrane filtration, electrodialysis, and photocatalysis [7,8,9,10]. However, to date, adsorption-based methods have proven to be the most effective and economical in removing toxic metal ions from wastewater [11]. In recent years, various materials of natural origin, agricultural waste, or industrial by-products have been tested as sorbents for removing toxic metal ions; however, only polymer sorbents revealed high adsorption capacity, good regeneration, and good selectivity for certain metal ions. Especially, functional polymers have made it possible to remove a wide range of metals due to the presence of complexing groups in the polymer structure. In particular, resins having carboxyl, phosphonic, or sulfonic functional groups have proved to be of great interest for the sorption of cationic species [12]. However, these resins do not have a significant adsorption capacity, while the regeneration of efficient chelating resins requires a strong acid desorbing agent, such as 4 M HCl in the case of glycidyl methacrylate-based polymer resins [13] or 1 M HNO_3_ in the case of cross-linked polyzwitterionic acid [14]. In the presented research, the precursors of the pyridinium moieties that were introduced onto the polymer surface are *N*-decyloxy-1-(pyridnyl)ethanimines with high potential as metal extractants from neutral and acidic aqueous solutions (Fe(III) from HCl [15], Cu(II) and Cu(I) from chloride and sulfate solution [16,17,18], Pb(II) from acidic chloride–nitrate solution [19,20], and Zn(II) from HCl solution [21]). These compounds show exceptionally high metal ion removal efficiency in liquid–liquid and membrane systems [22,23]. For example, using the pseudo-emulsion-based hollow fiber strip dispersion technique (PEHFSD), *N*-decyloxy-1-(pyridin-3-yl)ethanimine showed much greater potential as the Pd(II) carrier than the commercial extractant (Alamine 308) [23]. The specific properties of those compounds result from the presence in structure of both alkoxyimine and pyridine or pyridinium active sites after quaternization, which through various mechanisms can react with different metals species, e.g., the alkoxyimine group can bind a metal cation through O and N atoms, while the pyridinium cation shows the anion-binding affinity. The high extraction potential of these compounds results not only from the presence of groups capable of coordinating metals, but also from their amphiphilic nature, which enables the interaction in either polar or nonpolar solvent. Moreover, these compounds are also characterized by high stability, which guarantees their long-term use even after contact with strong acidic solutions. Therefore, the functionalization with this type of compounds should provide not only specific sorption properties but also stability, both chemical and thermal.

Owing to the above facts, the present study was undertaken to assess the sorption potential of novel resins fabricated through poly(vinylbenzyl chloride-co-divinylbenzene) and poly(vinylbenzyl bromide-co-divinylbenzene) quaternization of *N*-decyloxy-1-(pyridin-3-yl)ethaneimine and *N*-decyloxy-1-(pyridin-4-yl)ethaneimine. The research concerned the influence of the structure of precursor functional groups on the composition, morphology, and textural properties of the fabricated resins. In the scope of a sorption study, the effect of several parameters including pH, metal ion concentrations, contact time, and sorbet dosage were studied in detail and were discussed to evaluate the efficiency of Pb(II), Cd(II), Cu(II), Zn(II), and Ni(II) removal.

## 2. Materials and Methods

### 2.1. Chemicals

*N*-decyloxy-1-(pyridin-3-yl)ethaneimine (D3EI) and *N*-decyloxy-1-(pyridin-4-yl)ethaneimine (D4EI), obtained through a procedure described in [23,24] after purification by column chromatography (purities 99.9% determined by NMR), were used as the precursors of the functional groups. Poly(vinylbenzyl chloride-co-divinylbenzene) (VBC), and poly(vinylbenzyl bromide-co-divinylbenzene) (VBBr) beads fabricated by suspension polymerization according to procedures described in [25] were used as the polymer matrix (suspension polymerization, 5.0 mmol Cl/g resin and 4.7 mmol Br/g resin, respectively). Diluents such as toluene (99.9%), acetonitrile (99.9%), and methanol (99.9%) were purchased from POCh (Poland) and used as received. Lead nitrate (Pb(NO_3_)_2_, p.a.), copper(II) nitrate (Cu(NO_3_)_2_ × 6 H_2_O, p.a.), cadmium(II) nitrate (Cd(NO_3_)_2_ × 4 H_2_O, 99.997%), nickel(II) nitrate (Ni(NO_3_)_2_ × 6 H_2_O, p.a.), and zinc(II) nitrate (Zn(NO_3_)_2_ × 6 H_2_O, p.a.) were purchased from Sigma-Aldrich (Darmstadt, Germany). Standard solutions of metals as well as pH buffers were purchased from Merck KGaA (Germany). HCl (32%) was of analytical grade and was supplied by POCh (Poland). Ultra-pure water was obtained by using an Arium Pro DI purification system (Sartorius, Göttingen, Germany).

### 2.2. Instrumentation

Functionalization with the pyridine derivatives was confirmed via FTIR, NMR, XPS, and by elemental analysis. FT-IR analyses were carried out on a Vertex 70 spectrometer (Bruker, Ettlingen, Germany) in the range of IR 400–4000 cm^−1^ with a resolution of 2 cm^−1^ and using the KBr pellet method. ^13^C CP-MAS NMR spectra of the resins were recorded at a temperature of 23 °C on a Bruker AVANCE III 400 WB spectrometer. Elemental compositions of the fabricated resins were determined using a Vario ELIII element analyzer (Elementar Analysensysteme GmbH, Langenselbold, German). X-ray photoelectron spectroscopy was also used to determine elemental composition of the fabricated resins. The XPS analyses were carried out in a PHI VersaProbeII scanning XPS system (Physical Electronics Inc., Chanhassen, MN, USA) using monochromatic Al Kα (1486.6 eV) X-rays focused to a 100 µm spot. The photoelectron take-off angle was 45°, and the pass energy in the analyzer was set to 117.50 eV for survey scans and 46.95 eV to obtain high energy resolution spectra for the C 1s, N 1s, Cl 2p, Br 3d, and O 1s regions. A dual beam charge compensation with 7 eV Ar^+^ ions and 1 eV electrons was used to maintain a constant sample surface potential regardless of the sample conductivity. XPS depth profiling was realized with an Ar gas cluster ions beam (Ar-GCIB). The average size of a cluster was 4000 atoms, beam energy was set to 10 keV, and beam current was 11 nA. Deconvolution of spectra was carried out using PHI MultiPak software (v.9.9.0.8). Spectrum background was subtracted using the Shirley method.

Thermogravimetric analysis was used to study the thermal stability of the prepared sorbents. Measurements were performed on a TG 209 F3 Tarsus analyzer (NETZSCH-Geratebau GmbH, Germany) in the temperature range 30–600 °C. In each measurement, 10 mg of sample was placed in an Al_2_O_3_ crucible and was analyzed with a heating rate of 10 °C/min under nitrogen atmosphere (flow of protective gas 10 mL/min and purge gas 20 mL/min). The differential scanning calorimetry (DSC) measurements were performed using a DSC1 instrument (Mettler-Toledo, Greifensee, Switzerland). Before measurement, 10 mg of the prepared material was placed in an aluminum pan and sealed with a pan lid. The sample was then studied in the temperature range 25 to 200 °C with a heating/cooling rate of 20 °C/min under argon atmosphere (20 mL∙min^−1^). The morphology of the functionalized polymer surface was also analyzed using an FEI Quanta 250 FEG scanning electron microscope. The SEM images were taken for secondary electrons in the low vacuum mode at a pressure of 70 Pa, at an accelerating voltage of 20 kV. The low-temperature N_2_ sorption at −196 °C was applied to evaluate the textural properties. Nitrogen adsorption isotherms were measured at 77 K on an ASAP2020 volumetric analyzer manufactured by Micromeritics, Inc. (Norcross, GA, USA). Prior to measurement, the samples were degassed for 12 h at 80 °C. The average pore size was determined using the Barrett–Joyner–Halenda (BJH) method. An atomic absorption spectrometer (AAS; ContrAA 300, Analytik Jena, Jena, Germany) was used for the measurements of metal ion concentrations in aqueous samples. Determination of the static contact angle was carried out using the automated drop tensiometer Tracker™ equipped with a monochrome CCD camera (Teclis Scientific, Civrieux d’Azergues, France). For this purpose, the fabricated materials were subjected to the pelleting process (200 mg samples were pressed under a pressure of 5 bar for 60 s). The drop profile was determined using a producer software program that analyses the drop profile and fits it to models based on the Young–Laplace equation, and on this basis, the contact angles were determined. The contact angle was measured at least three times on different sites of the pallet surface. Each data point reported is the average of triplicate measurements.

### 2.3. Resins Fabrication

**Figure 1:**
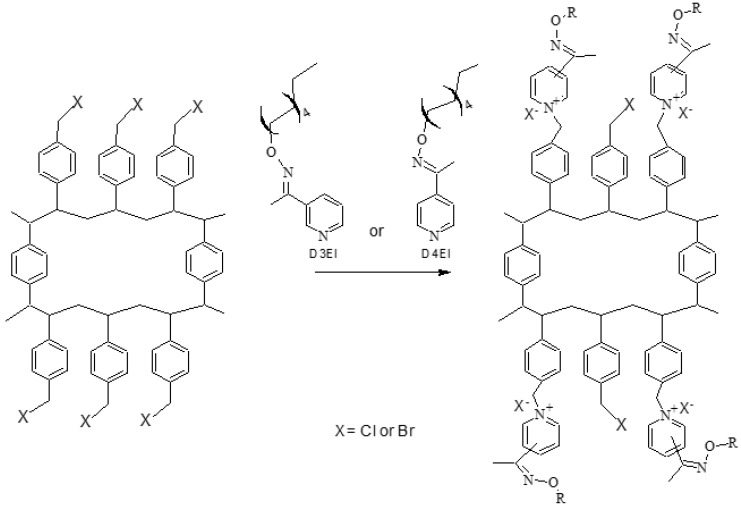
Scheme of the functionalization with D3EI and D4EI.

The synthesized poly(vinylbenzyl chloride-co-divinylbenzene) (VBC) and the poly(vinylbenzyl bromide-co-divinylbenzene) (VBBr) beads reacted with equimolar amount of *N*-decyloxy-1-(pyridin-3-yl)ethaneimine (D3EI) and *N*-decyloxy-1-(pyridin-4-yl)ethane-imine (D4EI) using toluene as reaction medium. The quaternization was carried out for 14 days at 60 °C and at nitrogen atmosphere in a 250 mL glass reactor equipped with a hot-plate–magnetic stirrer. The resulting resins were filtered, followed by Soxhlet extraction (methanol, 12 h) to remove unreacted D3EI and D4EI. The purified resins were dried at 80 °C under vacuum (0.01 mbar) and analyzed to confirm the functionalization. The scheme of the synthesis of the studied sorbents is presented in Figure 1.

### 2.4. Sorption Studies

Sorption experiments were carried out by employing the batch method by mixing a 0.1 g portion of the sorbent with 100 mL of an aqueous solution of Pb(II), Cu(II), Cd(II), Zn(II), and Ni(II) with variable concentrations of each metal salt. The adsorption was studied at a different pH (1–5 for Cu(II), Cd(II), and Zn(II), and 1–6 for Pb(II) and Ni(II), time (1–350 min) and metal ion concentration (50–200 mg/L), and at different temperatures (25–45 °C). The mixtures were shaken using a temperature-controlled shaker (KS 4000 ic control, IKA) at 240 rpm for a desired period of time. After finishing sorption or desorption, the mixture was passed through a filter (Whatman, 0.47 µm), and the metal ion concentrations in the supernatant were determined by atomic absorption measurements. Each experiment was replicated three times, and the results were averaged. The working metal concentrations were prepared by the appropriate dilution of stock solutions. The pH of each of the solution was adjusted to the desired value using a T5 Excellence titrator fitted with a pH electrode (Mettler Toledo, Columbus, USA). The desorption experiment was conducted for a selected resin using different aqueous solutions of HCl. In order to determine the reusability of the resins, consecutive adsorption–desorption cycles were repeated five times. The quantity of metal ions adsorbed per gram of the resin was calculated using Equation (1):

(1) $$q=\frac{\left(C_{0}-C_{t}\right)\cdot V}{m}$$

where C_0_ and C_t_ (mg/L) are concentrations of the metal ions before and after contact, respectively, of phases at time t. V is volume of aqueous phase used (L), and m is mass of the sorbent used (g).

The experimental data were also used to evaluate kinetics and equilibrium of the sorption. The pseudo-first order, pseudo-second order, Elovich, and intra-particle diffusion models were used for testing dynamic experimental data [26,27,28,29]. The interactions between the sorbent and the metals ions were analyzed using adsorption isotherm models. In the studies, the equilibrium data were modelled using the Freundlich, Langmuir, and Dubinin–Radushkevich isotherm models [30,31]. All used models and appropriate parameters are presented in Table 1.

## 3. Results and Discussion

### 3.1. Structural and Thermal Characterization of Resins

The synthesis of VBC-D3EI, VBC-D4EI, VBBr-D3EI, and VBBr-D4EI resins through VBC and VBBr quaternization of *N*-decyloxy-1-(pyridin-3- or -4-yl)ethaneimine was verified by the FTIR spectra of the final quaternized products, in comparison with the spectra of the initial copolymers. The FT-IR spectra revealed the appearance of a new strong band assigned to C=N vibrations of the pyridinium nitrogen. In the case of VBC-D3EI and VBC-D4EI, the bands were observed at 1631 and 1639 cm^−1^, respectively, while in the case of VBBr-D3EI and VBBr-D4EI, they were observed at 1623 and 1643 cm^−1^, respectively. This band was much more intense when the imine substituent was located at position 4 of the pyridine ring. An increase in intensity was also observed at 1022 cm^−1^, which also confirmed the presence of pyridinium moieties in the structure of the polymer. The imine C=N vibrations were also observed on each spectrum; however, the band was much less intense and was observed at 1608 or 1610 cm^−1^ for the D3EI and D4EI derivatives, respectively. Regardless of location of the imine substituent in the pyridine ring and structure of the polymer matrix, the spectra also revealed an increase in intensity of peaks at 1508–1510 and 1442–1448 cm^−1^, which were attributed to C=C vibrations, and the appearance of new peaks assigned to C=N-O bonds (at 1155 cm^−1^ (VBC-D4EI), 1161 cm^−1^ (VBC-D3EI), 1168 cm^−1^ (VBBr-D4EI), and at 1157 cm^−1^ (VBBr-D3EI). Moreover, similar to the unmodified VBC, the spectra of VBC-D3EI and VBC-D4EI also indicated the presence of the peaks attributed to stretching vibrations of the chloromethylene group (at 1261 cm^−1^ and at 802 cm^−1^). In the case of VBBr, VBBr-D3EI, and VBBr-D4EI, peaks observed at 1071 cm^−1^ and 707 cm^−1^ were attributed to stretching vibrations –CH_2_Br and C–Br.

The CP-MS ^13^C-NMR spectrum of the fabricated resins showed peaks at chemical shifts of 14.9, 26.7, 39.4, and 45.9 ppm, due to aliphatic CH_3_, CH_2_, and CH groups. In the case of VBC-D3EI and VBC-D4EI, carbon atoms of the CH_2_Cl appeared at 64.1 and 64.2 ppm, respectively. Moreover, after modification with D3EI, the signals assigned to aromatic rings were observed at 128.4, 145.8 (increased in intensity), and 152.3 ppm, and the imine C=N appeared at 120.9 ppm. After modification with D4EI, the signals assigned to the aromatic ring observed for VBC at 128.4 ppm increased, reaching a maximum at 122.1 ppm, and new signals appeared at 144.1 and 153.4 ppm. The imine C=N was hidden by pyridine CH atoms. The CP-MS ^13^C-NMR spectrum of VBBr after quaternization of D3EI and D4EI resulted in chemical shifts of 14.8, 22.6, 29.5, 39.4, and 45.9 ppm, which were assigned to aliphatic CH_3_, CH_2_, and CH groups, and signals at 65.3 and at 65.1 ppm corresponding to CH_2_Br attached to the pyridine nitrogen of D3EI and D4EI, respectively. The pyridine C and CH atoms in VBBr-D3EI were indicated at 127.2, 135.5, and 145.4 ppm, while in VBBr-D4EI they were indicated at 123.7, 144.3, and at 153.1 ppm. The imine C=N was observed at 122.1 ppm.

Based on elemental analyses, the degree of the quaternization and the amounts of the 3- or 4-[1-(decyloxyimine)ethyl]pyridinium groups incorporated in the polymer matrix were calculated. The results are presented in Table 2 and reveal that the involvement of the functional groups in the polymer matrix depend on the structure of the pyridinium moiety and type of counter anion. More effective functionalization was observed for moieties bearing the imine substituent at the 4-position of the pyridine ring than in the case of the 3-position, and quaternization with the chloromethylene moieties occurred more readily than with the bromethylene. The counter-anion had a significant influence on the quaternization reaction. Metzger [32] pointed out that an increase in the basicity of the leaving group shifted the transition state of the quaternization to the products; thus, the more labile group is bromide. It can be assumed that the location of the substituent in the 3-position and Br^−^ with ion radii 0.196 caused a spatial hindrance, limiting the concentration of functional groups in the polymer matrix.

**Figure 2:**
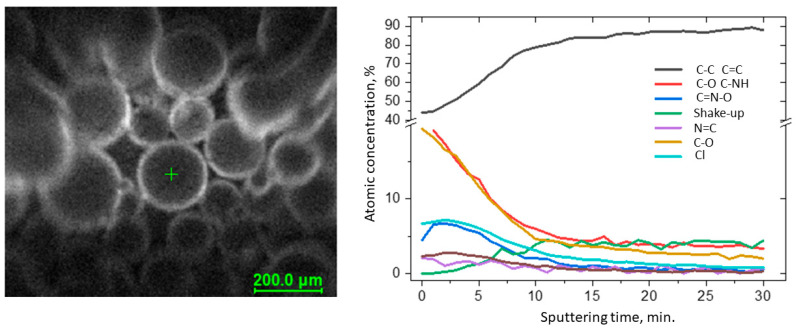
Results of the XPS depth profiling study of VBC-D4EI.

Full characterization of the functionalized surface required measuring the depth profiles of chemical composition (Figure 2). The C 1s spectra were resolved into four components by peak fitting. The first line centered at 284.8 eV arose from aliphatic and aromatic carbon bonds, the second line lying at 286.5 eV indicated the presence of C-NH bonds, and the third line centered at 289.0 eV indicated the presence of the imine N–C–O bonds [33]. The line at approx. 292 eV was identified as the π → π* shake-up line. This state resulted from 1s photoelectrons emitted from carbon atoms that excited an aromatic ring before leaving the sample surface and is indicative of the presence of aromatic C=C and C=N structures (sp^2^) [34]. The occurrence of the shake-up peak confirmed the presence of pyridine functionalities on the surface. During sputtering, the C–NH and N–C–O bond components decreased, whereas C–C and C=C increased. The spectra collected in the N 1s region show a single peak at 401.0 eV attributed to pyridine [35,36] and imine nitrogens [37], but for these bands, the changes were not observed during sputtering, probably due to their insignificant participation in the resin structure.

The specific surface area and pore structure of the fabricated resins were also investigated through low-temperature nitrogen sorption. Results showed that the specific surface area varied between 28.4 and 39.1 m^2^/g and strongly depended on the amount of functionalities incorporated onto the surface of the resins (Table 2). Moreover, regardless of the tested resins, the N_2_ adsorption curves were a type-IV according to the IUPAC classification, indicating the mesoporous nature of the sorbent. Moreover, for all fabricated products, the H3 hysteresis loop was observed, indicating the presence of narrow slit-like pores, as well as particles with internal voids of irregular shape and broad size distribution.

**Figure 3:**
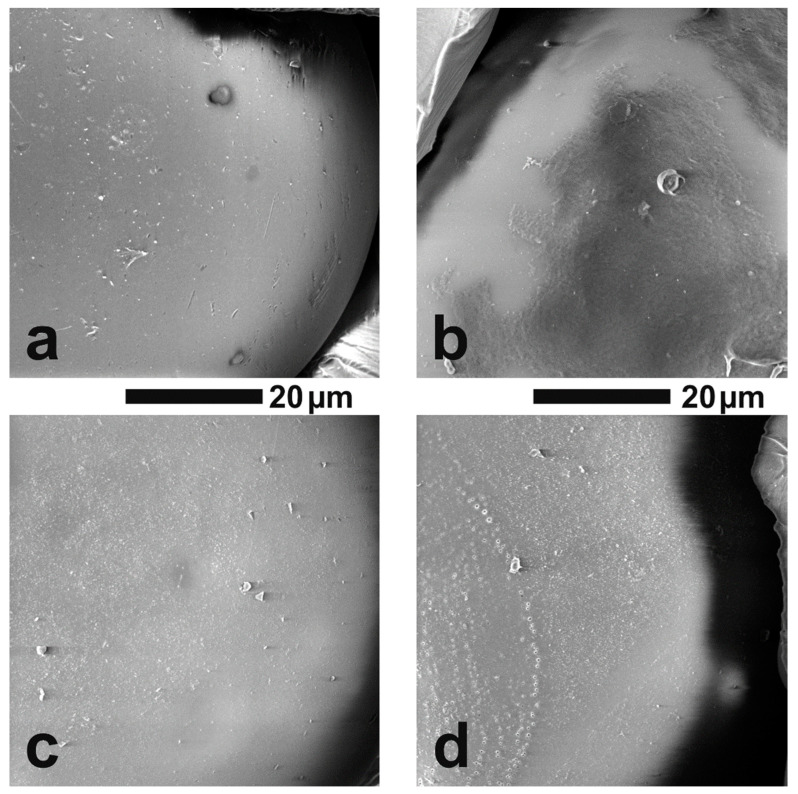
The grain surface of the tested resins at a magnification of 5000×: (**a**) VBC-D3EI, (**b**) VBC-D4EI, (**c**) VBBr-D3EI, (**d**) VBBr-D4EI.

The surface morphologies of VBC and VBBr series resin particles were observed by SEM, and representative images are shown on Figure 3. It could be observed that the surface of all analyzed materials was homogeneous and smooth. It had rare defects and a small number of pores with nanometer diameters. There were no significant differences between the individual materials. Moreover, as in the case of the unfunctionalized polymer matrix, it was observed that more than 90 percent of the imaged grains were in the size range of 175–300 μm (diameter), and the shape of the grains was perfectly spherical, and there was an occasional defect that looked similar to a dent from an adjacent ball.

The measurement of the contact angle provides information on the wettability of water on the resin surface. The results obtained show that the carried-out functionalization of VBC and VBBr with D3EI and D4EI caused poor surface wetting (θ > 90°) (Table 2). A correlation between the structure of functional groups (the substituents’ location and type of the counter anion) and the hydrophilicity of the sorbents was also observed. It was shown that the functionalization with 3-substituted pyridine yielded lower contact angle values than that observed for 4-analogue. Similarly, the presence of Br^−^ as the counter anion made the surface more hydrophobic than in the presence of Cl^−^.

**Figure 4:**
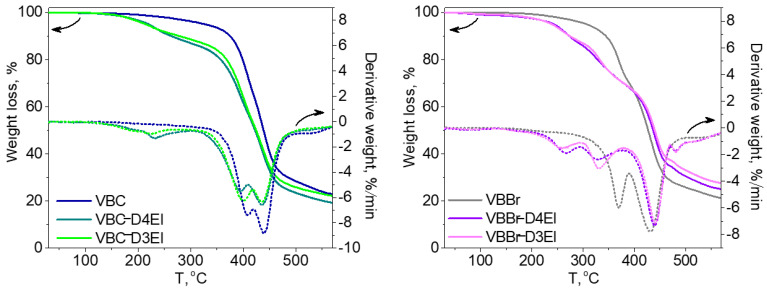
TG and DTG curves of unmodified and modified copolymers: (**a**) VBC and (**b**) VBBr with D3EI and D4EI.

The thermal properties of the synthesized sorbents were investigated using thermogravimetric analysis (TGA) and differential scanning calorimetry (DSC) methods. In Figure 4, TGA and DTG curves of two types of the studied copolymers VBBr and VBC, as well as products of their modification with two types of functionalities (D3EI and D4EI), are presented. As can be seen, their thermal degradation was a multistep process. The investigated copolymers had three stages of thermal decomposition, while sorbents, after modification, had more decomposition stages, which could be associated with grafted structures resulting from the modification. The first one, which occurred below 100 °C, is related to solvent evaporation. These residual amounts of solvent could be absorbed by the polymer grains during their synthesis and purification. The next two stages are the decomposition of the copolymer: the pendant groups, and then the crosslinked network. The beginning of the thermal decomposition of the copolymers was determined as the temperature of the onset of weight loss and is presented in Table 3. Generally, unmodified sorbents have higher thermal stability (above 300 °C) than modified sorbents (below 250 °C). Comparison with the literature data [25] showed that the obtained sorbents had a higher thermal resistance than previously obtained by us modified VBC and VBBr copolymers with substituents K4.10, K3.10, Ox3.10, and Ox4.10, as well as the commercial Lewatit TP 207 [38] or Amberlyst 15 [39] resin. Additionally, the VBC copolymer was characterized by higher thermal stability (T_onset_ = 382 °C) than VBBr copolymer (T_onset_ = 336 °C), by about 50 °C. Despite these differences in the thermal resistance of unmodified copolymers, the introduction of substituents led to materials with similar resistance on thermal degradation. Thus, the thermal resistance was significantly influenced by the substituents introduced into the copolymers during the modification. However, after modification, VBBr-based sorbents had slightly greater thermal stability than those obtained on the basis of VBC. No phase transitions, related to, e.g., glass transition temperature, were observed in the DSC thermograms. This may be due to the highly cross-linked structure of the copolymers. On the other hand, the residual solvent evaporation transition was observed. The heat of this transition was calculated from the thermograms, as well as the temperature at the maximum of the peak of this transition, and the results are presented in Table 3. The heat of evaporation of the solvent in the case of VBBr copolymers increased after modification of their structure, while it decreased in the case of VBC copolymers. This characteristic may be related to the sorption capacity of the synthesized materials. Thus, it may decide on their use as metals ions sorbents.

### 3.2. Sorption Studies

#### 3.2.1. Effect of pH

**Figure 5:**
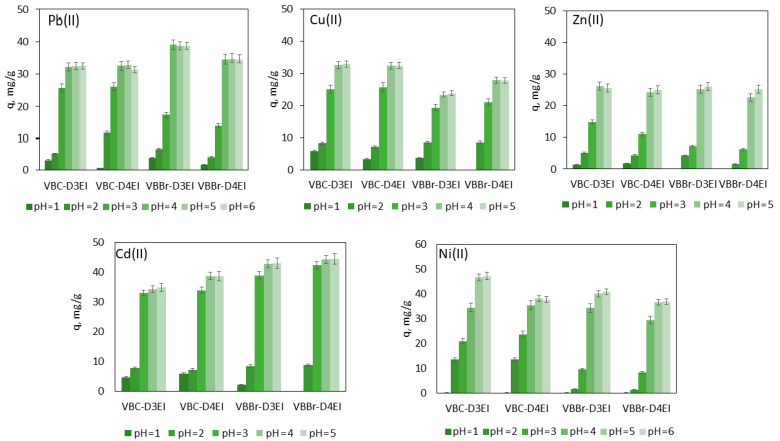
Effect of pH on metal ion adsorption onto the fabricated resins (metals ion concentration, 50 mg/L; sorbent dosage, 0.1 g; V, 100 mL).

Pb(II), Cu(II), Cd(II), Zn(II), and Ni(II) can be components of waste solutions from various industrial sectors, which can be characterized by different pH values, while the sorption efficiency depends on the physicochemical properties of the sorbent and changes in the chemical speciation of the metals removed, which may vary depending on the pH of the aqueous feed solution. Hence, the influence of pH on metal removal is significant. The results of tests carried out for Pb(II) and Ni(II) in the range pH 1–6, and Cu(II), Zn(II), and Cd(II) in the range pH 1–5 are presented in Figure 5. The differences resulted from the different solubilities of the metal salts at a given pH. The data obtained showed that regardless of the type of resins and removed metal ions, at pH of 1 the sorption almost did not occur, or the amount of metal removed did not exceed the value of 5 mg/g. The further increase in the pH value increased the efficiency of the sorption; however, in the case of Zn(II) and all considered resins, as well as in the case of Ni(II) and the resin series VBBr, the increase was the lowest and did not exceed q = 5 mg/g. For Cd(II), a rapid increase in the sorption efficiency was noted up to pH 3, and above this value the amount of metal ions removed remained constant. For Pb(II), Cu(II), and Zn(II), the increase was observed up to pH 4, and similar to Cd(II), further increases in value of pH did not cause any further changes in sorption efficiency. However, for Ni(II), the increase in the sorption efficiency was observed up to pH 5. It was also observed that the most effective sorption was observed for Ni(II) and all tested resins. The results also showed that the Zn(II) sorption was not affected by the type of resin, the resins series VBBr were less effective towards Cu(II), and in the case of the Pb(II) removal, the VBBr resins turned out to be much more efficient than the VBC resins.

#### 3.2.2. Effect of Time and Kinetics

**Figure 6:**
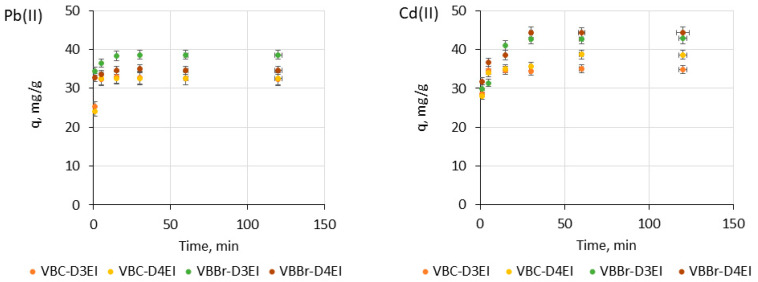
Effect of time on Pb(II) and Cd(II) adsorption onto the fabricated resins (metal ion concentration, 50 mg/L; sorbent dosage, 0.1 g; V, 100 mL).

The effect of contact time on the metal uptake by the fabricated resins was also studied. The experiment was carried out at a pH of 5 to avoid the formation of insoluble metal hydroxides. The results obtained for Pb(II) and Cd(II) are presented in Figure 6. The results indicated a rapid increase of binding between ions and the resins until a state of equilibrium was reached. In the case of Pb(II) sorption, the equilibrium was achieved at 5 min (the resins series VBC) and 15 min (the resins series VBBr) of shaking. A similar effect was observed for the Cd(II) sorption; however, using VBBr-D4EI, achieving equilibrium required much longer contact time (30 min). It is also important that no significant change in the adsorption capacity was observed with further increases in the adsorption time. In the case of Zn(II) and Ni(II), and using the resin series VBC, the equilibrium was reached at 5 min of shaking, using VBBr-D3EI at 15 min, but sorption with VBBr-D3EI required 30 min to achieve equilibrium. In the case of Cu(II) ions, the effect of contact time of both phases on the metal uptake is comparable to that observed for Pb(II).

**Figure 7:**
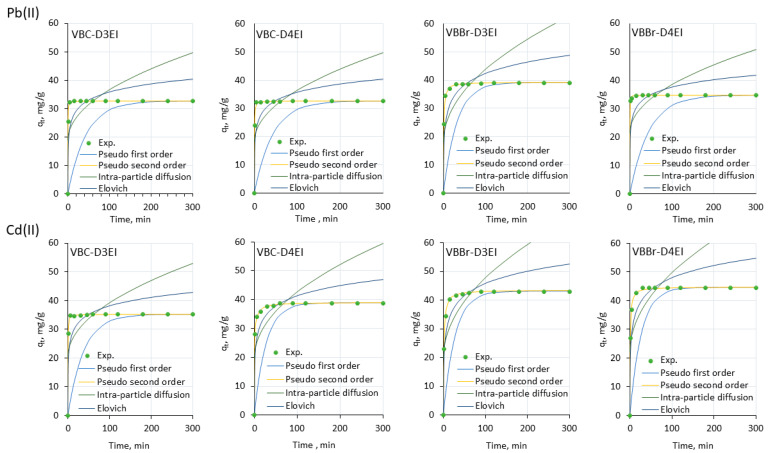
Experimental data of adsorption kinetic and non-linear fitting of kinetic models for adsorption of Pb(II) and Cd(II) onto resin surfaces (metal ion concentration, 50 mg/L; sorbent dosage, 0.1 g; V, 100 mL, pH 5).

**Figure 8:**
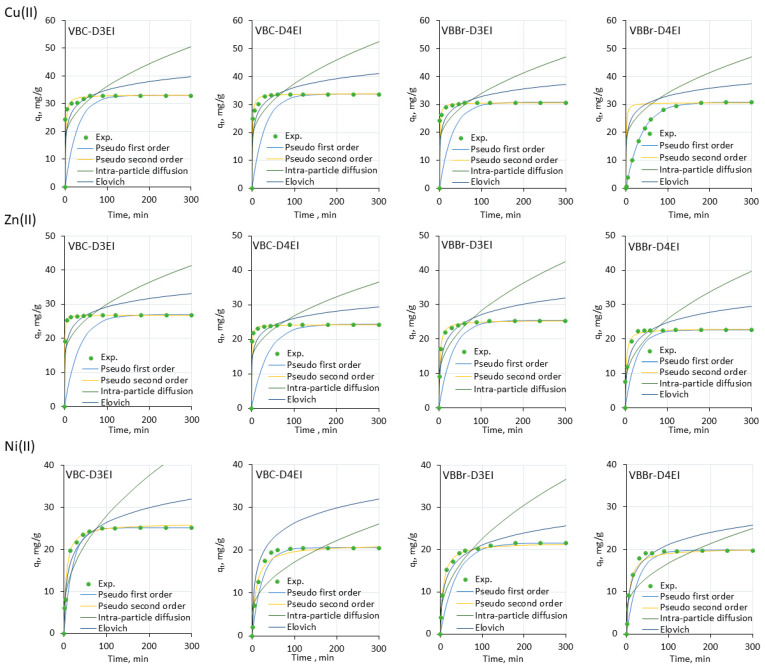
Experimental data of adsorption kinetics and non-linear fitting of kinetic models for adsorption of Cu(II), Ni(II), and Zn(II) onto resin surfaces (metal ion concentration, 50 mg/L; sorbent dosage, 0.1 g; V, 100 mL, pH 5).

In the next stage of the research, the pseudo-first-order, pseudo-second-order, Elovich, and Intra-particle diffusion kinetic models were fitted to the experimental data using non-linear regression [26,27,28,29]. The calculated parameters of the kinetic models are listed in Table S1, but the line graphs of the fit results are presented in Figure 7 and Figure 8.

The results presented indicate that the values’ correlation coefficients depended on the structure of the tested resins (location of the substituent at the pyridine ring and type of counter anion) as well as on the type of metals ions. Among the different models, the pseudo-second order model showed a very good fit with the experimental kinetic data for all sorption systems (R^2^ = 0.999–1.000). Moreover, the calculated q_e_ values were close to those obtained from the experimental data. The result was characteristic of chemical sorption, in which metals ions interact with the functionalized surface through chemical reaction of the electrophilic cationic metals species with the nucleophilic pyridinium and the imine moieties. The estimated kinetic constants also revealed that sorption of Pb(II) onto VBC-D4EI was the fastest (0.472 g/mg·min) with respect to other resins. Analyzing all the other fittings, it was found that intra-particle diffusion gave the worst fitting as demonstrated by the low values of the determination coefficients obtained both for the resins series VBC and VBBr-D3EI (R^2^ = 0.502–0.990). In the case of VBBr-D4EI, the worst fitting was obtained using the pseudo-first order model (R^2^ = 0.353–0.760).

#### 3.2.3. Adsorption Isotherms

**Figure 9:**
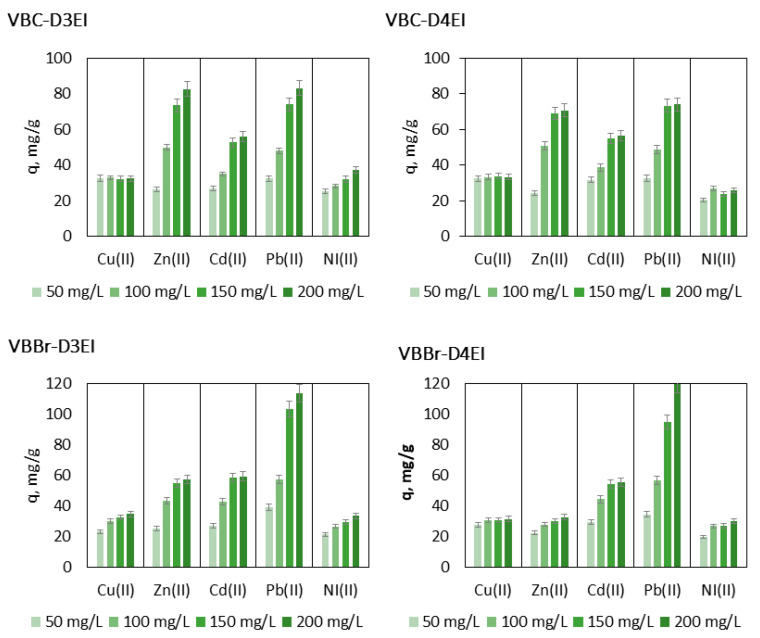
Effect of shaking time on sorption of Pb(II), Cu(II), Ni(II), Zn(II), and Cd(II) onto resin surfaces (metal ions concentrations, 50−200 mg/L; sorbent dosage, 0.1 g; V, 100 mL, pH 5).

To optimize the metal removal process, it is important to obtain information on the mobility of metal ions from the aqueous phase to the solid phase. The initial concentrations of solute had a large influence on the removal and capacity of the sorbent; therefore, the effect of the initial metal ion concentrations on sorption efficiency was also studied. The test was evaluated using the initial Pb(II), Cu(II), Cd(II), Zn(II), and Ni(II) concentrations ranging from 50 to 200 mg/L, at a constant temperature 23 °C, shaking time 120 min., resin dosage 0.1 g, and volume of the aqueous solution 100 mL. The obtained results (Figure 9) indicated that in the case of Cu(II), only using VBBr-D3EI, the metal uptake increased with the increase in the initial Cu(II) concentration. In the case of other resins, that effect was not observed, and the maximum amount of the removed ions was achieved just after contact with the solution containing Cu(II) in 50 mg/L. In the case of other metal ions, sorption increased with the increase in the initial concentration of metal ions, and for almost all tested systems, the sorption capacity was not reached, even after contact with the 200 mg/L solution. Experimental data also revealed that among the tested resins, the most efficient sorbent of Zn(II) and Ni(II) was VBC-D3EI (q = 86.7 mg/g and 37.1 mg/g, respectively), VBBr-D3EI was the most efficient for Cd(II) (q = 61.4 mg/g), while VBBr-D4EI was the most efficient for Pd(II) (q = 119.8 mg/g).

For the selected resins and the appropriate metals ions, the effect of the resin dosage on the capacity was also tested (resin dosage per liter of aqueous solution 0.2–1.0 g/L). It was observed that a five-fold increase in the volume of the aqueous solution resulted in a decrease in the sorption efficiency; however, the amount of adsorbed metals ions on the resin surface increased. For example, in the case of the Pb(II) sorption with VBBr-D4EI, the values of q increased from 119.8 mg/g to 378.1 mg/g; in the case of Cd(II) and VBBr-D3EI as the sorbent, the increase was from 61.4 mg/g to 174.9; while in the case of the Zn(II) sorption with VBC-D3EI, the results were comparable for both dosages.

Various isotherm models (Langmuir, Freundlich, and Dubinin–Radushkevich) were used to describe the equilibrium of the metal ions removal, and to predict the theoretical capacities of Pb(II), Cu(II), Ni(II), Zn(II), and Cd(II on the resin surface. The conducted adsorption data analysis concluded that regardless of type of resins and metals removed, the Langmuir isotherm was found to be the best model to present the equilibrium data (R^2^ = 0.993–1.000) (isotherms parameters presented in Table S2). This model describes a metal removal process based on its interaction with the surface. Thus, higher values of the Langmuir constant imply a greater affinity of the removed metal ions for the resin surface.

It could be observed that strong interaction did not necessarily result in significant uptake capacity. For example, sorption of Ni(II) with VBBr-D4EI exhibited K_L_ value 0.05 L/mg and uptake capacity 33.7 mg/g, but sorption of Pb(II) with VBBr-D4EI exhibited K_L_ value 0.01 L/mg and uptake capacity 296.4.7 mg/g. For other resins and metals removed, the maximum monolayer adsorption capacities were as follows: 117.7, 71.7, 156.1, and 39.3 mg/g for Pb(II), Cd(II), Zn(II), and Ni(II) sorption with VBC-D3EI, respectively; 90.9, 65.5, 20.18, and 26.0 mg/g for Pb(II), Cd(II), Zn(II), and Ni(II) sorption with VBC-D4EI, respectively; and 155.5, 83.8, 78.6, 38.1, and 37.7 mg/g for Pb(II), Cd(II), Zn(II), Cu(II), and Ni(II) sorption with VBBr-D3EI, respectively. Moreover, the values of parameter R_L_ (separation factor) were in the range of 0–1, indicting the adsorption as favorable.

#### 3.2.4. Comparison with Commercial Sorbents

The estimated adsorption abilities were also compared with sorption properties of the commercial ion-exchange (Lewatit TP 208, Duolite GT-73) and the chelating resin (Amberlite IRA743). From the presented comparison (Table 4), it can be seen that the adsorption capacity of Pb(II) on VBBr-D4EI was much higher than any other sorbents, while VBC-D4EI had significantly higher adsorption capacity for Zn(II). In the case of sorption of the remaining tested metal ions, the obtained capacities significantly exceeded the values indicated for Lewatit TP 208, Lewatit TP 260, and Lewatit CNP 80, while Duolite GT-73 was competitive in this respect.

#### 3.2.5. Adsorption from Mixture of Metal Ions

Adsorption performance was evaluated by the experiments carried out using the VBBr-D4EI resin and two synthetic waste solutions: gold mining effluent containing 85.0 mg/L Pb(II), 2.6 mg/L Cu(II), and 0.4 mg/L Zn(II) [43]; and electrolytic zinc residue percolate containing 173.2 mg/L Pb(II), 123.8 mg/L Cd(II), 236.4 mg/L Zn(II), 7.0 mg/L Cu(II), and 35.9 mg/L Mg(II) [44]. In this test, 100 mg of the resin was dispersed into 100 mL of the aqueous solution, and the mixture was shaken for 120 min. On the basis of the obtained results, it was found that the sorption of the metal ions from their mixture depended on the composition of the aqueous phase. In the case of the synthetic gold mining effluent, in which Pb(II) was the dominant metal ion, only Pb(II) was removed (78 mg/g), but Zn(II) and Cu(II) remained in the aqueous solution. In contrast, sorption from the electrolytic zinc residue percolate, in which Pb(II), Cd(II), and Zn(II) were at a comparable concentration, showed that Pb(II) was removed by 54.3% (q = 94.1 mg/g) and Cd(II) by 5.2% (q = 6.4 mg/g). Other ions such as Zn(II), Cu(II), and Mg(II) remained in the aqueous solution. These results indicated the high potential of VBBr-D4EI for the removal of Pb(II) from wastewater.

### 3.3. Desorption Studies

Based on the obtained results, the various concentrations (0.01–1 M) of HCl were tested as desorbing agents for Pb(II), Cu(II), Cd(II), Zn(II), and Ni(II) from the loaded VBC-D4EI. Before the tests, VBC-D4EI was contacted with the aqueous solutions containing 200 mg/L of each metal ions. The desorption was conducted for 15 min, at a temperature of 25 °C, using 1 g of the loaded VBC-D4EI and 100 mL of the HCl solution. The results showed that 0.1 M HCl gave efficient desorption (94–99%), while 0.01 M HCl ensured desorption at 82–89% (Table 5). However, using 0.01 M HCl as the desorption agent, the losses in the sorption efficiency were the lowest.

## 4. Conclusions and Future Perspectives

The novel resins VBC-D3EI, VBC-D4EI, VBBr-D3EI, and VBBr-D4EI were fabricated through poly(vinylbenzyl chloride-co-divinylbenzene) (VBC), and poly(vinylbenzyl bromide-co-divinylbenzene) (VBBr) quaternization of *N*-decyloxy-1-(pyridin-3-yl)ethaneimine and *N*-decyloxy-1-(pyridin-4-yl)ethaneimine, and tested as sorbents of Pb(II), Cd(II), Cu(II), Zn(II), and Ni(II) from the aqueous solutions. The NMR, FT-IR, and elemental analysis confirmed the polymers’ functionalization and allowed for the determination of the degree of quaternization and the number of functional groups introduced onto the resins’ surfaces. In addition, the XPS depth profiling study provided detailed information on the chemical compositions of the resins. It was also shown that the structure of the pyridinium group and the type of counter ion affected the hydrophilicity of the surface and their textural properties. The sorption studies also showed that the amount and structure of the functional group on the resin surface significantly influenced the sorption efficiency of the metal ions. Functionalization with the 4-substituted pyridine enabled the incorporation of more groups on the polymer surface than with the 3-substituted pyridine, and sorption of the metals occurred with higher efficiency. Additionally, the presence of bromide as the counter ion made the resins more efficient. Other factors were pH, shaking time, and metal ion concentration. The results obtained also showed that regardless of the structure of the functionalities, the metal sorption was very fast, and equilibrium was reached even within 5 min of shaking. Additionally, the pseudo second order model was found to be the most suitable model for fitting kinetics data. The adsorption process was best described through the Langmuir isothermal adsorption model, and the predicated adsorption capacities of Pb(II), Zn(II), Cd(II), Cu(II), and Ni(II) were 296.4, 201.8, 83.8, 38.1, and 39.3 mg/g, respectively. In the case of Pb(II) and Zn(II), the capacities are superior to the adsorption capacities of the commercial resins. The conducted sorption–desorption tests confirmed that the adsorbed metal can be effectively removed with 0.01 M HCl, and the regenerated resin can be reused without significant loss in the sorption properties. Those desorption properties and the simple regeneration method make the novel resins attractive and promising adsorbents for removal of Pb(II), Cd(II), Cu(II), Zn(II), and Ni(II) from aqueous solutions. Moreover, the VBBr-D4EI resin also showed increased efficiency in the Pb(II) removal from wastewater, and in the absence of Cd(II) also high selectivity.

The results showed high sorption potential of the obtained resins, and future work should be focused on increasing the selectivity, e.g., by modification within the alkoxy group.

## Figures and Tables

**Table 1:** Kinetic and isotherm models with equations used to describe sorption process.

Kinetic Model	Equation		Parameters
Pseudo-first order	$q_{t}=q_{e}\;\left(1-e^{-k_{1}t}\right)$	(2)	q_e_—amount of metal ions removed at quilibrium q_t_—amount of metal ions removed at time t k_1_—pseudo-first-order model k_2_—pseudo-second order rate constant α -initial sorption rate constant β—desorption constant k_ip_—intra-particle diffusion rate constant C—thickness of boundary layer.
Pseudo-second order	$q_{t}=\frac{k_{2}\cdot q_{e}^{2}}{1+k_{2}\cdot q_{e}}$	(3)	
Elovich	$q_{t}=\frac{1}{\beta }\ln\left(\alpha \beta t\right)$	(4)	
Intra-particle diffusion	$q_{t}=k_{\mathrm{ip}}t^{\frac{1}{2}}+C$	(5)	
**Isotherm Model**	**Equation**		**Parameters**
Langmuir	$q_{e}=\frac{q_{m}K_{L}C_{e}}{1+K_{L}C_{e}}$	(6)	C_e_—equilibrium concentration of adsorbed metal ions K_L—_Langmuir equilibrium constant R_L_—dimensionless separation factor K_F_—Freundlich equilibrium constant K_DR_—Dubinin–Radushkevich equilibrium constant q_m_—maximum sorption capacity n—heterogeneity factor
Freundlich	$R_{L}=\frac{1}{1+K_{L}C_{e}}$	(7)	
	$q_{e}=K_{F}{\;C}_{e}^{\frac{1}{n}}$	(8)	
Dubinin–Radushkevich	$q_{e}={\;q}_{m}e^{-K_{\mathrm{DR}}\;{\left[\mathrm{RT}\;\ln\left(1+\frac{1}{C_{e}}\right)\right]}^{2}}$	(9)	

**Table 2:** Results of elemental, textural, and wettability analysis of the fabricated resins.

Sample	Degree of Quaternization %	C_p_^1^ mmol/g	Pore Size nm	Specific Surface Area m^2^/g	Contact Angle Degree
VBC	-	5.0	-	-	-
VBC-D3EI	90.1	2.1	6.6	33.3	105.4
VBC-D4EI	80.9	2.2	5.8	39.1	110.7
VBBr	-	4.7	-	-	-
VBBr-D3EI	84.2	1.8	7.8	28.4	123.2
VBBr-D4EI	75.9	1.9	6.2	35.7	125.3

C_p_^1^, the concentration of pyridinium group.

**Table 3:** Thermal properties of prepared resins.

	T_onset_, °C	Hp*, J/g	Tp*, °C
VBBr	336	10.1	80.0
VBBr-D3EI	241	30.5	90.9
VBBr-D4EI	244	36.4	94.9
VBC	382	23.1	83.9
VBC-D3EI	174	4.7	66.0
VBC-D4EI	213	6.7	69.3
Lewatit TP 207	80		
Amberlyst 15	150		

T_onset_—temperature of sorbents decomposition determined from TGA measurements. Hp*—the heat of solvent evaporation. Tp*—the temperature at the maximum of the peak of Hp determined from DSC thermograms.

**Table 4:** Comparison of capacities towards Cu(II), Cd(II), and Zn(II) of the selected adsorbents (pH 4–6). Bold is highlight the best results.

Adsorbent	Adsorption Capacity, mg/g					Ref.
	Pb(II)	Cu(II)	Cd(II)	Zn(II)	Ni(II)	
Lewatit CNP 80	73.4	10.2	4.9	20.3	18.9	[40]
Duolite GT-73	122.3	61.6	105.7	55.6	56.9	[41]
Lewatit TP 260	1.9	70.1	-	3.5	0.5	[42]
Lewatit TP 208	1.8	71.1	-	2.7	0.6	[42]
Amberlite IRA743	1.5	36.0	-	0.3	0.1	[42]
VBBr-D4EI	**296.4**	31.6	65.4	34.5	33.7	This work
VBC-D4EI	90.9	33.2	65.5	**201.8**	26.0	

**Table 5:** Results of desorption tests from the loaded VBC-D4EI.

Metal	Desorption Agent HCl mol/L	Desorption %	Efficiency Loss ^1^ %
Pb(II)	0.01	88.9	3.0
	0.1	97.8	5.3
Cu(II)	0.01	80.1	4.7
	0.1	99.2	6.9
Cd(II)	0.01	82.7	3.1
	0.1	99.5	6.4
Ni(II)	0.01	83.2	3.7
	0.1	99.2	7.4
Zn(II)	0.01	87.9	4.7
	0.1	99.4	7.4

^1^ Efficiency loss calculated after five sorption–desorption cycles.

## Data Availability

Not applicable.

## Supplementary Materials

The following supporting information can be downloaded online. Table S1: Kinetic parameters of Pb(II), Cd(II), Zn(II), Cu(II), and Ni(II) sorption kinetics on the fabricated resins, as determined by fitting with the selected models; Table S2: Isotherm parameters of different models for sorption of Pb(II), Cd(II), Zn(II), Cu(II), and Ni(II) sorption kinetics on the fabricated resins, as determined by fitting with the selected models.

## Sample Availability

Samples of all synthetized resins, namely *N*-decyloxy-1-(pyridin-3-yl)ethaneimine and *N*-decyloxy-1-(pyridin-4-yl)ethaneimine, are available from the authors.
