# Supplementary material for: Poly(vinylbenzyl Pyridinium Salts) as Novel Sorbents for Hazardous Metals Ions Removal
Source: Molecules. 2022 Mar 6;27(5):1723. doi: 10.3390/molecules27051723 (PMC8911724; doi:10.3390/molecules27051723)
Supplement: Supplementary file 1 [file molecules-27-01723-s001.zip › molecules-1628281-supplementary.pdf]

Table S1. Kinetic parameters of Pb(II), Cd(II), Zn(II), Cu(II) and Ni(II) sorption kinetics on the fabricated resins as determined by fitting with the selected models.

Isotherm model	VBC – D3EI					VBC-D4EI					VBBr-D3EI					VBBr – D4EI				
	Pb(II)	Cd(II)	Zn(II)	Cu(II)	Ni(II)	Pb(II)	Cd(II)	Zn(II)	Cu(II)	Ni(II)	Pb(II)	Cd(II)	Zn(II)	Cu(II)	Ni(II)	Pb(II)	Cd(II)	Zn(II)	Cu(II)	Ni(II)
q_{exp} (mg/g)	32.8	35.3	26.8	33.0	25.1	32.7	38.8	24.3	33.7	20.5	39.2	43.2	25.4	30.7	21.6	34.8	44.6	22.7	30.8	19.8
Pseudo-first order																				
k_1(min^{-1})	0.023	0.027	0.026	0.035	0.066	0.024	0.037	0.023	0.034	0.055	0.029	0.037	0.031	0.034	0.037	0.022	0.037	0.039	0.027	0.049
q_e (mg/g)	32.8	35.3	26.8	33.0	25.1	32.7	38.7	24.3	33.8	20.6	39.2	43.2	25.4	30.7	21.6	34.8	44.8	22.7	30.8	19.8
R^2	0.908	0.837	0.719	0.876	0.854	0.996	0.839	0.981	0.866	0.695	0.936	0.935	0.952	0.855	0.905	0.535	0.661	0.760	0.742	0.693
Pseudo-second order																				
k_2 (g/mg min)	0.327	0.152	0.127	0.028	0.018	0.159	0.024	0.096	0.039	0.014	0.024	0.019	0.019	0.046	0.012	0.472	0.037	0.039	0.065	0.016
q_e (mg/g)	32.4	35.2	26.9	33.1	25.7	32.6	38.9	24.2	33.8	20.9	39.2	43.4	25.5	30.8	21.7	34.7	44.8	23.1	30.7	20.1
R^2	1.000	1.000	1.000	0.999	1.000	1.000	0.999	0.999	0.999	0.999	0.999	0.999	0.999	0.999	0.999	1.000	0.999	0.999	0.999	0.999
Intra-particle diffusion																				
k_{ip2} (mg/g min$^{0.5}$)	1.74	1.67	1.55	1.96	2.75	1.82	2.31	1.21	2.04	1.37	2.09	2.79	1.96	1.82	2.40	1.69	2.77	1.92	1.77	1.28
C	18.9	20.5	14.5	16.7	4.85	18.3	19.6	14.5	17.0	5.5	21.6	19.8	8.6	15.8	2.6	21.6	21.9	6.6	16.3	5.8
R^2	0.502	0.912	0.734	0.835	0.727	0.990	0.782	0.943	0.792	0.687	0.941	0.823	0.713	0.751	0.826	0.610	0.756	0.735	0.674	0.652
Elovich																				
α (mg/g min)	254.0	318.3	125.9	134.9	24.0	195.8	156.5	398.3	127.1	24.2	381.2	105.3	19.8	136.5	14.9	728.8	143.0	13.1	157.4	29.9
β (g mg)	0.245	0.231	0.279	0.229	0.229	0.236	0.194	0.372	0.219	0.328	0.220	0.162	0.225	0.248	0.271	0.263	0.163	0.232	0.250	0.345
R^2	0.620	0.831	0.929	0.977	0.914	0.951	0.943	0.972	0.958	0.913	0.828	0.936	0.942	0.880	0.957	0.873	0.926	0.931	0.850	0.912

Table S2. Isotherms parameters of different models for sorption of Pb(II), Cd(II), Zn(II), Cu(II) and Ni(II) sorption kinetics on the fabricated resins as determined by fitting with the selected models.

**Isotherm model**	**VBC – D3EI**				**VBC-D4EI**				**VBBr-D3EI**					**VBBr – D4EI**			
	Pb(II)	Cd(II)	Zn(II)	Ni(II)	Pb(II)	Cd(II)	Zn(II)	Ni(II)	Pb(II)	Cd(II)	Zn(II)	Cu(II)	Ni(II)	Pb(II)	Cd(II)	Zn(II)	Ni(II)
Langmuir																	
Q_m (mg/g)	117.7	71.7	156.1	39.3	90.9	63.5	201.8	26.0	155.5	83.8	78.6	38.1	37.7	296.4	65.4	34.5	33.7
K_L (L/mg)	0.02	0.03	0.01	0.06	0.03	0.05	0.01	0.09	0.03	0.02	0.02	0.06	0.04	0.01	0.04	0.06	0.05
R^2	0.997	0.999	1.000	0.999	0.993	0.999	0.995	0.998	0.999	0.999	1.000	0.992	0.998	0.999	0.997	0.999	0.999
Freundlich																	
K_F ($mg^{1-(1/n)}/gL^n$)	7.6	7.4	4.2	13.4	10.2	13.9	2.4	14.1	12.5	6.7	5.7	11.7	9.4	4.6	11.2	12.2	8.8
N	1.95	2.38	1.54	5.08	2.39	3.51	1.37	9.29	1.98	2.16	2.10	4.66	4.01	1.32	3.00	5.29	4.09
R^2	0.978	0.969	0.994	0.993	0.965	0.987	0.975	0.940	0.966	0.974	0.983	0.985	0.994	0.994	0.962	0.991	0.943
Dubinin-Radushkevich																	
Q_m(mg/g)	75.2	53.6	77.3	34.7	67.8	52.5	70.7	23.9	99.9	58.0	53.8	33.6	31.8	105.5	53.5	30.7	29.5
K_{DR} (mol^2/kJ^2)	$4.7\cdot10^{-5}$	$6.6\cdot10^{-5}$	$7.5\cdot10^{-5}$	$3.5\cdot10^{-5}$	$3.9\cdot10^{-5}$	$3.0\cdot10^{-5}$	$1.2\cdot10^{-4}$	$2.3\cdot10^{-5}$	$2.1\cdot10^{-5}$	$6.8\cdot10^{-5}$	$8.0\cdot10^{-5}$	$4.3\cdot10^{-5}$	$5.5\cdot10^{-5}$	$4.7\cdot10^{-5}$	$4.2\cdot10^{-5}$	$4.0\cdot10^{-5}$	$6.3\cdot10^{-5}$
R^2	0.912	0.957	0.920	0.921	0.923	0.919	0.954	0.731	0.937	0.950	0.945	0.928	0.923	0.919	0.942	0.937	0.980
